# Comparative effectiveness of dulaglutide versus liraglutide in Asian type 2 diabetes patients: a multi-institutional cohort study and meta-analysis

**DOI:** 10.1186/s12933-020-01148-8

**Published:** 2020-10-09

**Authors:** Kai-Cheng Chang, Shih-Chieh Shao, Shihchen Kuo, Chen-Yi Yang, Hui-Yu Chen, Yuk-Ying Chan, Huang-Tz Ou

**Affiliations:** 1 Department of Pharmacy, Linkou Chang Gung Memorial Hospital, Taoyuan, Taiwan; 2 School of Pharmacy, Institute of Clinical Pharmacy and Pharmaceutical Science, College of Medicine, National Cheng Kung University, 1 University Road, Tainan, 701 Taiwan; 3 Department of Pharmacy, Keelung Chang Gung Memorial Hospital, Keelung, Taiwan; 4 Division of Metabolism, Endocrinology & Diabetes, Department of Internal Medicine, University of Michigan Medical School, Ann Arbor, MI USA; 5 Department of Pharmaceutical Materials Management, Chang Gung Medical Foundation, Taoyuan, Taiwan; 6 Department of Pharmacy, National Cheng Kung University Hospital, Tainan, Taiwan; 7 School of Pharmacy, College of Medicine, National Cheng Kung University, Tainan, Taiwan

**Keywords:** Dulaglutide, Liraglutide, Clinical effectiveness and meta-analysis

## Abstract

**Background:**

Head-to-head comparison of clinical effectiveness between dulaglutide and liraglutide in Asia is limited. This study was aimed to assess the real-world comparative effectiveness of dulaglutide versus liraglutide.

**Methods:**

We conducted a retrospective cohort study by utilizing multi-institutional electronic medical records to identify real-world type 2 diabetes patients treated with dulaglutide or liraglutide during 2016–2018 in Taiwan and followed up until 2019. Effectiveness outcomes were assessed at every 3 months in the 1-year follow-up. Propensity score techniques were applied to enhance between-group comparability. Significant differences in changes of effectiveness outcomes between treatment groups during the follow-up were examined and further analyzed using mixed-model repeated-measures approaches.

**Results:**

A total of 1512 subjects receiving dulaglutide and 1513 subjects receiving liraglutide were identified. At 12 months, significant HbA1c changes from baseline were found in both treatments (dulaglutide: − 1.06%, *p* < 0.001; liraglutide: − 0.83%, *p* < 0.001), with a significant between-group difference (− 0.23%, 95% confidence interval − 0.38 to − 0.08%, *p* < 0.01). Both treatments yielded significant declines in weight, alanine aminotransferase level, and estimated glomerular filtration rate from baseline (dulaglutide: − 1.14 kg, − 3.08 U/L and − 2.08 mL/min/1.73 m^2^, *p* < 0.01; liraglutide: − 1.64 kg, − 3.65 U/L and − 2.33 mL/min/1.73 m^2^, *p* < 0.001), whereas only dulaglutide yielded a significant systolic blood pressure reduction (− 2.47 mmHg, *p* < 0.001). Between-group differences in changes of weight, blood pressure, and liver and renal functions at 12 months were not statistically significant.

**Conclusions:**

In real-world T2D patients, dulaglutide versus liraglutide was associated with better glycemic control and comparable effects on changes of weight, blood pressure, and liver and renal functions.

## Introduction

Treatment with glucagon-like peptide-1 receptor agonists (GLP-1RAs) is recommended as a second- or third-line therapeutic option for patients with inadequately controlled type 2 diabetes (T2D) [1]. GLP-1RAs have exhibited promising glycemic efficacy, with additional favorable effects of weight loss, blood pressure reduction, and renal function preservation, and pose a low risk of hypoglycemia, all of which contribute to the desirable cardiovascular outcomes [2, 3].

Long-acting agents (liraglutide, dulaglutide, semaglutide) generally offer better glycemic control than a short-acting agent (lixisenatide, exenatide) [4]. Liraglutide and dulaglutide are the two commonly-used GLP-1RAs in East Asia. There are two head-to-head randomized, phase III trials of dulaglutide versus liraglutide. The international AWARD-6 trial [5] for patients recruited from nine countries and the other trial for Japanese patients [6] both demonstrated non-inferior HbA1c reduction between dulaglutide and liraglutide users at 26 weeks (between-group difference: − 0.06% in the AWARD-6 trial and − 0.10% in the Japanese trial, *p*_*non-inferiority*_ < 0.001). The Japanese trial further showed more favorable HbA1c reduction with dulaglutide versus liraglutide at 52 weeks (1.39% versus 1.19%; between-group difference: − 0.20%, *p* = 0.04) [7]. However, these trials had a relatively short follow-up period (26 weeks [5, 6]), small sample size (< 300 patients per treatment arm [5–7]), or a lack of generalizability due to the highly-selected populations [5–7].

Real-world evidence obtained from comparative effectiveness research can be used to complement evidence from trials through translating the efficacy of interventions in trials to the effectiveness of them in clinical practice among a broader spectrum of patient populations [8]. Evidence regarding the head-to-head comparative effectiveness of GLP-1RAs is critical for supporting clinical decisions and formulating healthcare reimbursement policies in real-world practice. There are few real-world studies, from Spain [9], Italy [10], Canada [11], United States [12, 13], India [14], and Scandinavian countries [15] with a limited number of study subjects (25–585 per treatment arm [9, 11–14]), patients with a specific comorbidity (i.e., solid organ transplant [13]), a short follow-up period (e.g., 13 weeks [14]), few clinical effectiveness measures (i.e., HbA1c only [12] or HbA1c, weight, and blood pressure only [9–11, 13, 14]), or specific severe clinical outcome events of interest (i.e., renal replacement therapy, death from renal causes, and hospitalization for renal events [15]). To date, there are no published real-word comparative effectiveness studies on GLP-1RAs for Western Pacific patients with T2D. We utilized multi-institutional electronic medical records (EMRs) to identify real-world Taiwanese T2D patients receiving GLP-1RAs for the comparative effectiveness of dulaglutide versus liraglutide on glycemic control, weight, blood pressure, and liver and renal functions.

## Methods

### Data source

The Chang Gung Research Database (CGRD) was utilized. It comprises de-identified individual EMRs of disease diagnoses, medical visits (outpatient, inpatient, and emergency room), pharmacy records, examination reports, and laboratory data from seven medical institutes throughout Taiwan, covering 1.3 million individuals (about 6% of Taiwan’s total population) [16]. Its validity for real-world pharmacoepidemiological studies is documented elsewhere [16–20].

### Study subjects

**Fig. 1:**
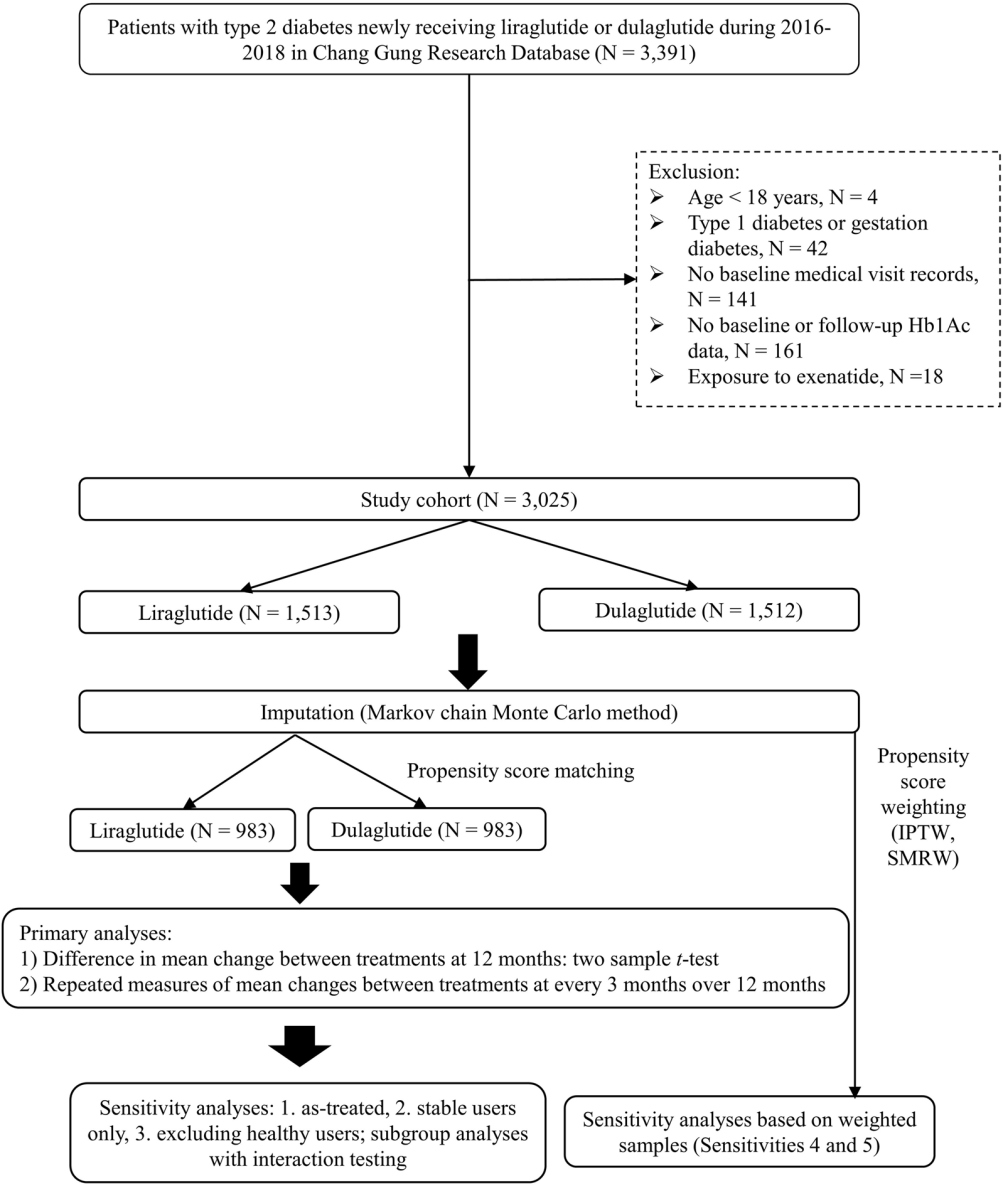
Flow chart of cohort selection and outline of analytic procedures. *IPTW* inverse probability of treatment weighting, *SMRW* standardized mortality ratio weighting

As shown in Fig. 1, we identified patients diagnosed with T2D (International Classification of Diseases, Ninth Revision [ICD-9] disease code of 250.X0 or 250.X2; ICD-10 disease code of E11) during 2015–2019. Then, we included patients aged 18 years or older and newly initiated on dulaglutide or liraglutide in 2016–2018. The first prescription date of dulaglutide or liraglutide was the index date. All patients were followed up from the index date until the end of 2019. The study subject identification period of 2016–2018 for GLP-1RAs users allowed for at least 1-year follow-up period. Patients were excluded if they were diagnosed with type 1 diabetes or gestation diabetes in the year before the index date, or treated with exenatide during the study period. To obtain sufficient data for patient baseline conditions, all study patients were required to have at least one clinic visit and one HbA1c record in the year before the index date.

### Study effectiveness outcomes

The primary outcome was the comparison of dulaglutide versus liraglutide on the HbA1c change from the index date (baseline) to 3, 6, 9, and 12 consecutive months in the 1-year follow-up. We identified HbA1c records within each 3-month interval and the HbA1c value closest to each corresponding assessment time point was used in the analyses. Body weight, systolic blood pressure (SBP), and liver (alanine aminotransferase [ALT]) and renal (estimated glomerular filtration rate [eGFR]) functions were also measured from baseline and every 3 months in the follow-up. We implemented multiple imputations using the Markov chain Monte Carlo method with an expectation maximization algorithm and combined 10 simulations to deal with missing data in the follow-up [21].

### Statistical analyses

Analytic procedures are outlined in Fig. 1. To minimize potential selection bias and enhance the comparability of study subjects between two treatment groups, we applied a propensity score (PS) matching procedure [22]. Propensity scores of study subjects were estimated using a multivariable logistic regression model based on various patient baseline characteristics listed in Table 1. We used the nearest-neighbor 1:1 PS matching with a caliper of 0.05 on the PS scale with 8-digit greedy matching [23].

**Table 1:** Characteristics of study patients before and after propensity score matching (PSM)

	Before PSM			After PSM		
	Dulaglutide (n = 1513)	Liraglutide (n = 1512)	SMD	Dulaglutide (n = 983)	Liraglutide (n = 983)	SMD
Demographics						
Age at the index date	57.6 ± 12.6	57.6 ± 13.6	< 0.01	57.0 ± 13.0	57.1 ± 13.3	< 0.01
Sex (male)	47.1%	50.6%	0.06	48.3%	47.6%	0.01
Biochemical tests in the year before the index date						
Weight (kg)	77.7 ± 18.2	77.1 ± 17.1	0.02	77.8 ± 17.6	77.5 ± 16.8	0.01
SBP (mmHg)	140.1 ± 20.2	139.8 ± 20.6	0.01	140.7 ± 19.9	140.2 ± 20.2	0.02
DBP (mmHg)	78.5 ± 12.0	77.1 ± 11.9	0.10	78.8 ± 12.0	78.2 ± 12.0	0.04
HbA1c (%)	9.3 ± 1.6	9.5 ± 1.7	0.14	9.3 ± 1.6	9.3 ± 1.5	0.02
Fasting plasma glucose (mg/dL)	177.9 ± 62.3	179.6 ± 69.8	0.02	179.3 ± 63.2	178.6 ± 67.4	0.01
Cholesterol (mg/dL)	175.6 ± 45.7	174.9 ± 45.3	< 0.01	176.4 ± 46.6	175.2 ± 43.1	0.02
HDL-C (mg/dL)	43.9 ± 12.3	42.6 ± 11.9	0.02	43.4 ± 12.7	43.6 ± 11.6	0.02
LDL-C (mg/dL)	96.3 ± 32.6	95.7 ± 34.0	0.08	97.1 ± 33.8	96.2 ± 32.9	0.01
Triglycerin (mg/dL)	207.9 ± 241.5	218.9 ± 240.1	0.04	214.9 ± 272.0	213.8 ± 247.5	< 0.01
eGFR (mL/min/1.73 m^2^)	81.5 ± 36.9	79.4 ± 38.3	0.06	82.4 ± 38.2	82.1 ± 35.6	< 0.01
ALT (U/L)	35.0 ± 29.4	34.5 ± 31.8	0.01	36.1 ± 30.2	35.4 ± 32.6	0.02
Prior comorbidities in the year before the index date						
aDCSI	1.8 ± 2.5	2.5 ± 2.9	0.24	2.0 ± 2.6	1.9 ± 2.3	0.02
CCI	1.8 ± 1.8	2.1 ± 2.0	0.14	1.9 ± 1.8	1.8 ± 1.8	< 0.01
Hypertension	65.0%	67.1%	0.04	65.3%	65.8%	0.01
Dyslipidemia	71.4%	71.6%	< 0.01	71.9%	72.5%	0.01
Ischemic heart disease	11.4%	19.6%	0.22	13.8%	14.3%	0.01
Heart failure	3.8%	6.4%	0.11	4.5%	3.9%	0.03
Cerebrovascular disease	6.6%	8.3%	0.06	6.9%	8.3%	0.05
Liver disease	18.7%	19.2%	0.01	18.4%	18.4%	< 0.01
COPD	2.3%	2.1%	< 0.01	2.2%	2.1%	< 0.01
CKD	11.6%	17.0%	0.15	12.4%	12.0%	0.01
Cancer	12.2%	10.9%	0.03	11.3%	11.2%	< 0.01
Prior exposure of co-medications in the year before the index date						
ACEI/ARB	60.2%	63.6%	0.07	61.3%	61.1%	< 0.01
Calcium channel blockers	22.2%	24.8%	0.06	22.0%	23.9%	0.04
β-blockers	28.9%	35.0%	0.13	29.8%	30.4%	0.01
Diuretics	15.0%	18.6%	0.09	14.8%	15.8%	0.02
Lipid-lowering agents	76.5%	76.3%	< 0.01	75.8%	75.6%	< 0.01
Nitrates	8.7%	15.1%	0.19	10.2%	10.0%	< 0.01
Digoxin	0.8%	0.8%	< 0.01	0.8%	0.6%	0.02
Antiplatelet	31.4%	36.4%	0.11	32.2%	32.2%	< 0.01
Anticoagulant	2.0%	3.2%	0.07	2.3%	2.3%	< 0.01
Antidepressant	8.4%	9.6%	0.04	9.1%	8.7%	0.01
Antipsychotic	4.2%	5.8%	0.06	4.8%	4.5%	0.01
NSAID	23.1%	24.3%	0.02	24.2%	24.5%	< 0.01
Concomitant GLAs at the index date						
Metformin	81.2%	67.8%	0.31	78.1%	78.0%	< 0.01
Sulfonylurea	70.6%	46.0%	0.51	61.1%	62.7%	0.03
DPP-4i	5.5%	4.5%	0.04	5.1%	5.1%	< 0.01
Thiazolidinedione	23.5%	10.8%	0.34	15.0%	14.8%	< 0.01
Alpha glucosidase inhibitors	18.8%	8.1%	0.31	12.0%	11.6%	0.01
Meglitinide	2.5%	4.2%	0.09	3.4%	2.7%	0.04
SGLT-2i	4.6%	2.4%	0.12	2.3%	3.2%	0.05
Medical specialty at the index date			0.29			0.05
Metabolism and endocrinology	81.8%	83.0%		83.3%	83.9%	
Cardiology	3.9%	9.0%		4.9%	5.4%	
Family medicine	1.5%	1.7%		1.5%	1.8%	
Other	12.8%	6.3%		10.3%	8.9%	
Hospital level at the index date			0.16			0.02
Medical centers	46.0%	40.7%		51.0%	51.0%	
Region hospitals	48.7%	55.5%		31.0%	30.3%	
Local hospitals	5.3%	3.8%		18.0%	18.7%	

A standardized mean difference (SMD) value of > 0.10 indicates a statistical difference in a given patient characteristic between the two drug groups. Index date refers to the first date of initiation of dulaglutide or liraglutide

*SMD* standardized mean difference, *SBP* systolic blood pressure, *DBP* diastolic blood pressure, *eGFR* estimated glomerular filtration rate, *ALT* alanine aminotransferase, *CCI* Charlson comorbidity index, *aDCSI* adapted Diabetes Complications Severity Index, *COPD* chronic obstructive pulmonary disease, *CKD* chronic kidney disease, *ACEI/ARB* angiotensin-converting enzyme inhibitors/angiotensin receptor blockers, *NSAID* non-steroidal anti-inflammatory drugs, *GLA* glucose lowering agents, *DPP-4i* dipeptidyl peptidase-4 inhibitors, *SGLT-2i* sodium-glucose transport protein-2 inhibitors

Primary analyses were based on an intention-to-treat (ITT) scenario where the loss to follow-up in the CGRD, death, or end of the 12-month follow-up, whichever came first, was censored. Analyses were divided into two parts. First, we used the paired *t*-test to estimate changes in clinical effectiveness at 12 months from baseline within each treatment group for assessing the within-group difference, and then used the two-sample *t*-test to determine the between-group difference in changes of clinical effectiveness at 12 months from baseline. Second, to consider time-varying changes in biomarkers (e.g., HbA1c) that were repeatedly assessed every 3 months during the follow-up, we performed a mixed-model analysis to consider treatment groups, assessment time points, and the interaction of treatment groups and assessment time points as fixed effects and individual patients as a random effect [24].

A series of sensitivity and subgroup analyses were conducted. First, to account for possible over-estimation of treatment effects in the ITT analyses where non-adherence to treatments was ignored, we performed the as-treated analysis where patients who switched away from or discontinued the use of a study drug were also censored, in addition to the censoring defined in the ITT analyses (Sensitivity 1). Second, to avoid potential confounding from short-term or accidental use of GLP-1RAs, we performed analyses where only stable users were included (Sensitivity 2). Stable users were defined as patients who had at least three consecutive refills of dulaglutide or liraglutide with any gaps between two consecutive refills of less than 90 days [25]. Third, we performed analyses with adjustment for potential healthy user bias (Sensitivity 3). Specifically, the patients who received GLP-1RAs and also used a dipeptidyl peptidase 4 inhibitor (DPP-4i) or sodium glucose cotransporter 2 inhibitor (SGLT-2i) were identified as possible healthy users because the combined use of a GLP-1RAs with a DPP-4i or SGLT-2i is not reimbursed by Taiwan’s National Health Insurance program and patients have to pay out-of-pocket fees. Under this circumstance, patients who are willing to self-pay for more intensive treatments would be likely to be engaged in healthier behaviors. We re-ran the analyses using a subset of patients who did not use a DPP-4i or SGLT-2i in combination with GLP-1RAs to avoid potential healthy user bias. Fourth, to enhance the study generalizability through retaining study cohort patients as many as possible, we applied two PS weighting procedures, inverse probability of treatment weighting (IPTW) and standardized mortality ratio weighting (SMRW) [26] (Sensitivities 4 and 5). Specifically, the patients at the 5th to 95th percentiles of the distribution of PS were first trimmed to minimize potential residual confounding [27]. Then, for IPTW, dulaglutide users were weighted as the inverse of the estimated PS and liraglutide users were weighted as the inverse of 1 minus the estimated PS. For SMRW, dulaglutide users were given a weight of 1 and liraglutide users were given a weight based on the ratio of the estimated PS to 1 minus the estimated PS.

In subgroup analyses, the procedures that were performed in the primary analyses were applied to examine the treatment effects on study outcomes in subgroups according to a series of patient baseline characteristics, including HbA1c (≥ 9%, < 9%), age (≥ 65 years, < 65 years), eGFR (≥ 60 mL/min/1.73 m^2^, < 60 mL/min/1.73 m^2^), ALT (> upper normal limit [UNL], ≤ UNL), and body mass index (≥ 27 kg/m^2^, < 27 kg/m^2^). A two-tail *p*-value of less than 0.05 was considered statistically significant. Data were analyzed using SAS Enterprise Guide, version 7.1 (SAS Institute, Cary, NC, USA).

### Meta-analysis

We further performed a meta-analysis on clinical effectiveness (i.e., HbA1c, weight and SBP) of liraglutide vs. dulaglutide by pooling the results from prior studies and the present study. Two reviewers (Chang and Shao) independently searched studies from the PubMed and Embase from the inception of database to May 31, 2020 that reported the comparison of liraglutide and dulaglutide. The search strategy and key terms were listed in Additional file 1: Appendix Table S1. Effectiveness outcomes abovementioned were measured from 6 and 12 months of follow-up periods. We included both randomized control trials (RCTs) and observational studies without imposing any language restrictions. Data were presented as mean difference with 95% CIs. We conducted the random-effects model meta-analysis using the reverse invariance method. The statistical heterogeneity was assessed by the statistic *I*^2^. To minimize the heterogeneity of included studies, we further conducted subgroups analyses for meta-analysis of RCTs or observational studies only. Data were analyzed by Review Manager version 5.3 (Copenhagen: The Nordic Cochrane Centre, The Cochrane Collaboration, 2014).

## Results

### Baseline characteristics of study patients

1512 and 1513 patients newly-initiated on dulaglutide and liraglutide, respectively, were included for the analyses under PS weighting procedures (IPTW and SMRW; Fig. 1). After PS matching, 983 matched pairs of dulaglutide and liraglutide new users were identified. Additional file 1: Appendix Figure S1 illustrates the kernel density for the PS distributions of the treatment groups. Table 1 shows patients’ characteristics for dulaglutide and liraglutide users before and after the PS matching. All patients’ characteristics were comparable between treatment groups after the PS matching.

### Glycemic control

**Fig. 2:**
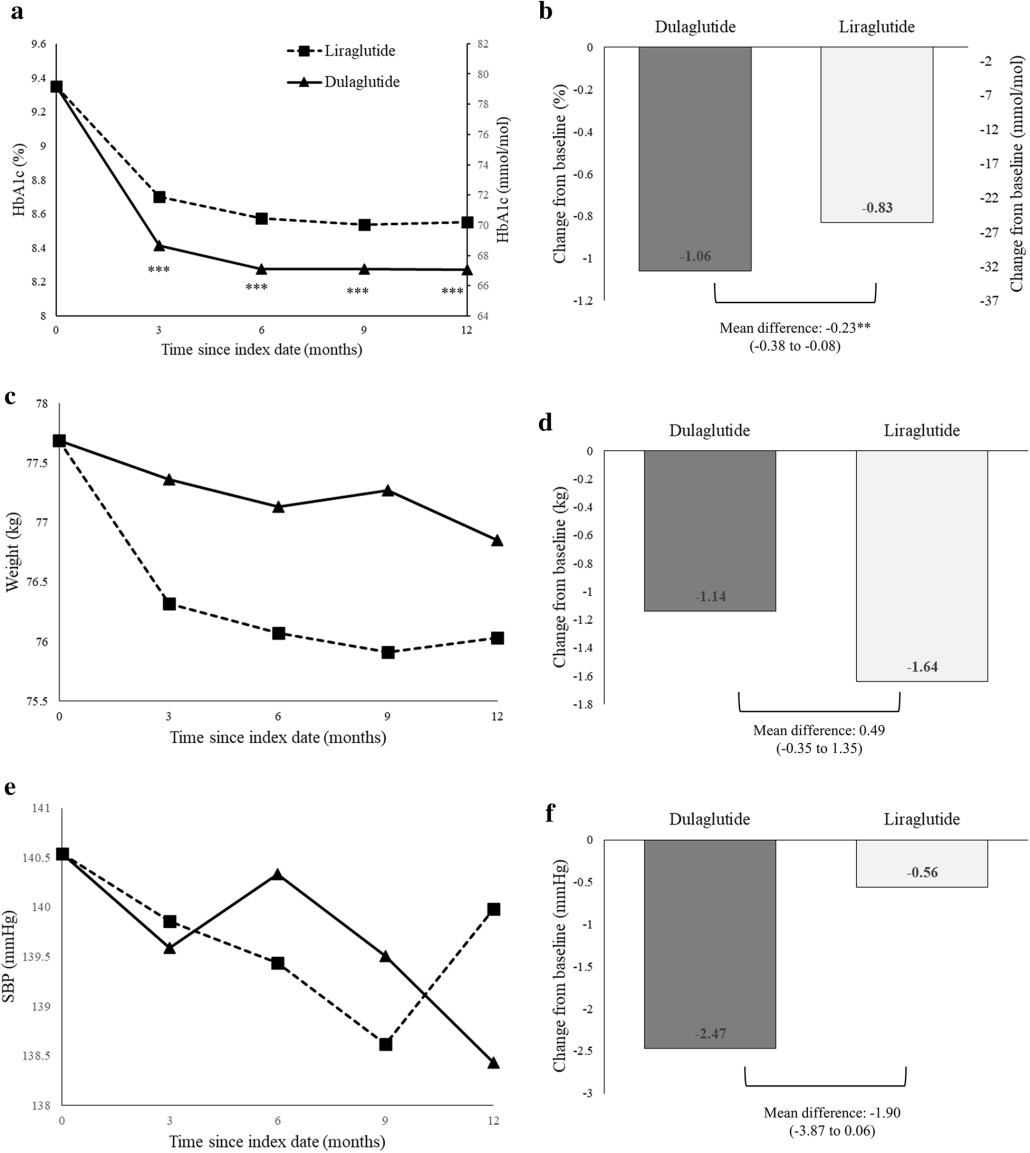

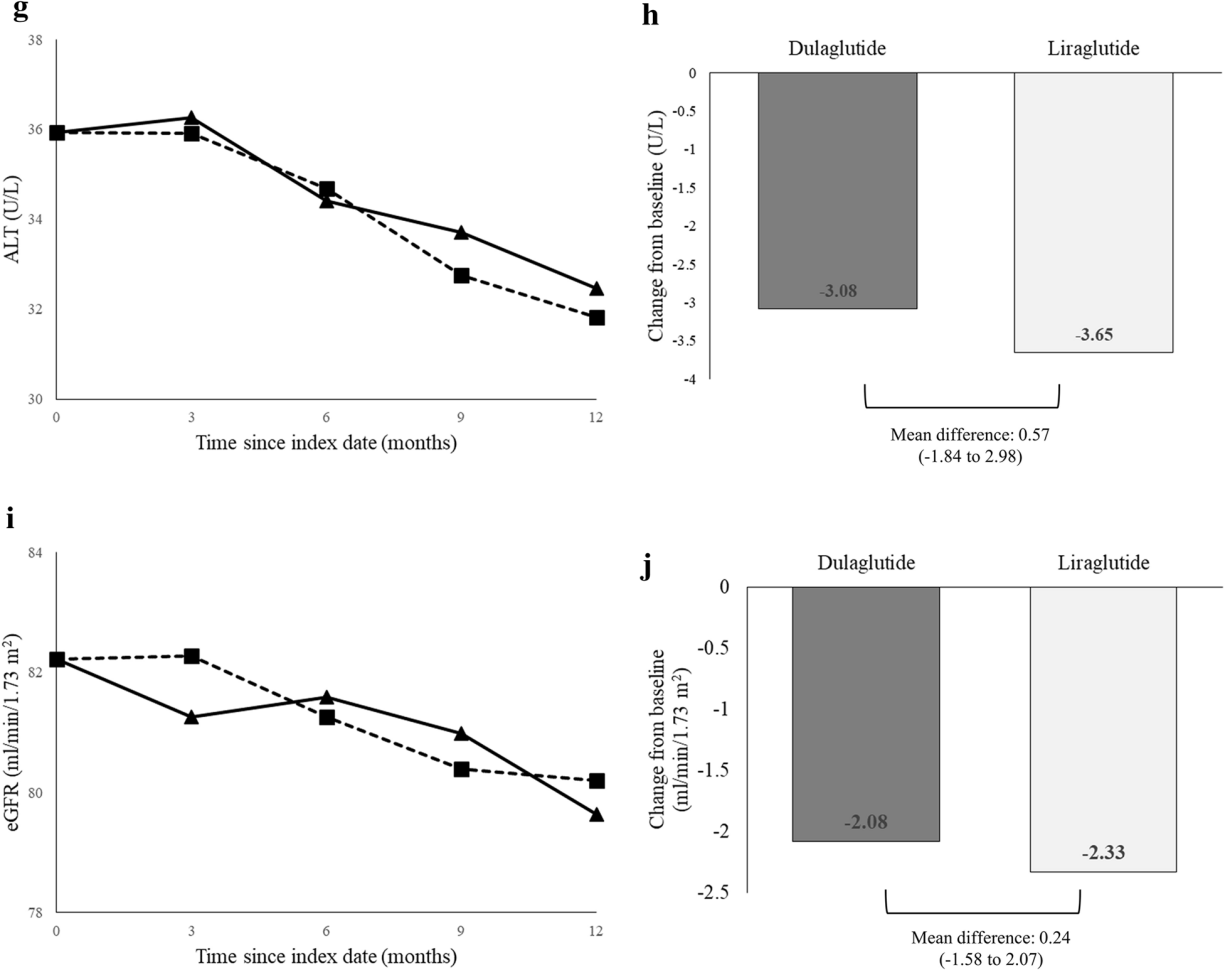
Changes in clinical effectiveness at every 3 months after initiation of dulaglutide or liraglutide (based on the propensity-score-matched sample). **a** HbA1c values from baseline to month 12, **b** change in HbA1c from baseline to month 12, **c** body weights from baseline to month 12, **d** change in body weight from baseline to month 12, **e** systolic blood pressure (SBP) from baseline to month 12, **f** change in SBP from baseline to month 12, **g** alanine aminotransferase (ALT) values from baseline to month 12, **h** change in ALT from baseline, **i** estimated glomerular filtration rates (eGFR) from baseline to month 12, and **j** change in eGFR from baseline to month 12. Index date refers to the first date of initiation of dulaglutide or liraglutide. *, **, and *** refer to *p* value < 0.05, < 0.01, and < 0.001, respectively

Based on the PS-matched sample, there was a statistically significant change in HbA1c at 12 months from baseline in each treatment group (dulaglutide: − 1.06% [standard deviation (SD): 1.70] and liraglutide: − 0.83% [SD: 1.61]), with a significant between-group difference in HbA1c reduction of − 0.23% (95% confidence interval [CI] − 0.38 to − 0.08%) (Table 2). Figure 2a, b show a large HbA1c reduction trend associated with dulaglutide versus liraglutide over the 1-year follow-up.

**Table 2:** Comparison of clinical effectiveness between dulaglutide and liraglutide users (based on the study sample after propensity score matching)

	Dulaglutide (n = 983)		Liraglutide (n = 983)		Dulaglutide versus liraglutide
	Baseline (SD)	Change from baseline (SE)	Baseline (SD)	Change from baseline (SE)	Mean difference (95% CI)
Analysis for the change before and after 12 months^a^					
HbA1c (%)	9.36 (1.66)	− 1.06 (0.05)***	9.33 (1.57)	− 0.83 (0.05)***	− 0.23 (− 0.38 to − 0.08)**
Weight (kg)	77.83 (17.69)	− 1.14 (0.30)***	77.53 (16.81)	− 1.64 (0.31)***	0.49 (− 0.35 to 1.35)
SBP (mmHg)	140.71 (19.94)	− 2.47 (0.69)***	140.24 (20.24)	− 0.56 (0.72)	− 1.90 (− 3.87 to 0.06)
ALT (U/L)	36.10 (30.23)	− 3.08 (0.82)***	35.47 (32.66)	− 3.65 (0.91)***	0.57 (− 1.84 to 2.98)
eGFR (mL/min/1.73 m^2^)	82.43 (38.21)	− 2.08 (0.69)**	82.13 (35.69)	− 2.33 (0.62)***	0.24 (− 1.58 to 2.07)
Analysis for repeated changes at every 3 months over 1 year^b^					
HbA1c (%)	9.36 (1.66)	− 1.09 (0.07)***	9.33 (1.57)	− 0.78 (0.07)***	-0.27 (− 0.43 to − 0.12)***
Weight (kg)	77.83 (17.69)	− 1.08 (0.35)**	77.53 (16.81)	− 1.41 (0.35)***	0.82 (− 0.76 to 2.41)
SBP (mmHg)	140.71 (19.94)	− 2.36 (0.93)*	140.24 (20.24)	− 0.31 (0.93)	− 1.55 (− 3.52 to 0.40)
ALT (U/L)	36.10 (30.23)	− 3.72 (1.63)*	35.47 (32.66)	− 3.85 (1.66)*	0.65 (− 3.01 to 4.31)
eGFR (mL/min/1.73 m^2^)	82.43 (38.21)	− 2.49 (1.06)*	82.13 (35.69)	− 2.09 (1.05)*	− 0.56 (− 3.99 to 2.85)

*SD* standard deviation, *SE* standard error, *CI* confidence interval, *SBP* systolic blood pressure, *eGFR* estimated glomerular filtration rate, *ALT* alanine aminotransferase

*, **, and *** refer to *p*-value < 0.05, < 0.01, and < 0.001, respectively

^a^For assessing clinical outcomes before and after 12 months of treatment, the paired *t*-test was used to estimate changes in clinical effectiveness at 12 months within each treatment group, and the two-sample *t*-test was applied to test the between-group difference in the change of clinical effectiveness at 12 months

^b^For assessing repeated clinical outcome changes at every 3 months over the 1-year follow-up, mixed-model analysis was carried out

### Body weight loss, blood pressure control, and liver and renal functions

At 12 months, the changes in body weight, SBP, eGFR, and ALT from baseline in dulaglutide users were − 1.14 kg, − 2.47 mmHg, − 2.08 mL/min/1.73 m^2^, and − 3.08 U/L, respectively, and those in liraglutide users were − 1.64 kg, − 0.56 mmHg, − 2.33 mL/min/1.73 m^2^, and − 3.65 U/L, respectively (Table 2 and Fig. 2). Among these outcomes, both dulaglutide and liraglutide users had significant reduction in body weight, eGFR, and ALT at 12 months from baseline, while only dulaglutide users had significant SBP reduction. The between-group differences in the changes in body weight, SBP, eGFR, and ALT levels did not reach statistical significance. Similar results were observed in the mixed-model analyses (Table 2).

### Sensitivity and subgroup analyses

A series of sensitivity analyses show consistent results (Additional file 1: Appendix Table S2) with the primary analyses (Table 2). The results of subgroup analyses are summarized in Additional file 1: Appendix Table S3 and Figure S2. Generally, there was a consistent benefit of dulaglutide versus liraglutide on HbA1c and SBP across all subgroups. In contrast, there was some heterogeneous treatment effects for other outcomes across subgroups. For example, at 12 months, liraglutide use yielded greater body weight reduction compared to dulaglutide use among patients with age ≥ 65 years old (between-group difference in weight reduction: 1.30 kg, *p* < 0.05). Among patients with abnormal liver function (ALT > UNL), the ALT levels in both treatment groups significantly declined at 12 months (i.e., dulaglutide: − 19.13 U/L [SD: 36.31] and liraglutide: − 22.79 U/L [41.85], *p* < 0.001), despite no statistically significant between-group difference in the ALT change (3.65 U/L, 95% CI − 2.45 to 9.76). The baseline HbA1c level appears to be a significant modifier for the comparative effectiveness of dulaglutide versus liraglutide; the interaction terms of treatment group (dulaglutide versus liraglutide) and baseline HbA1c level (≥ 9% versus < 9%) across different study outcomes were statistically significant (Additional file 1: Appendix Figure S2).

### Meta-analysis

**Fig. 3:**
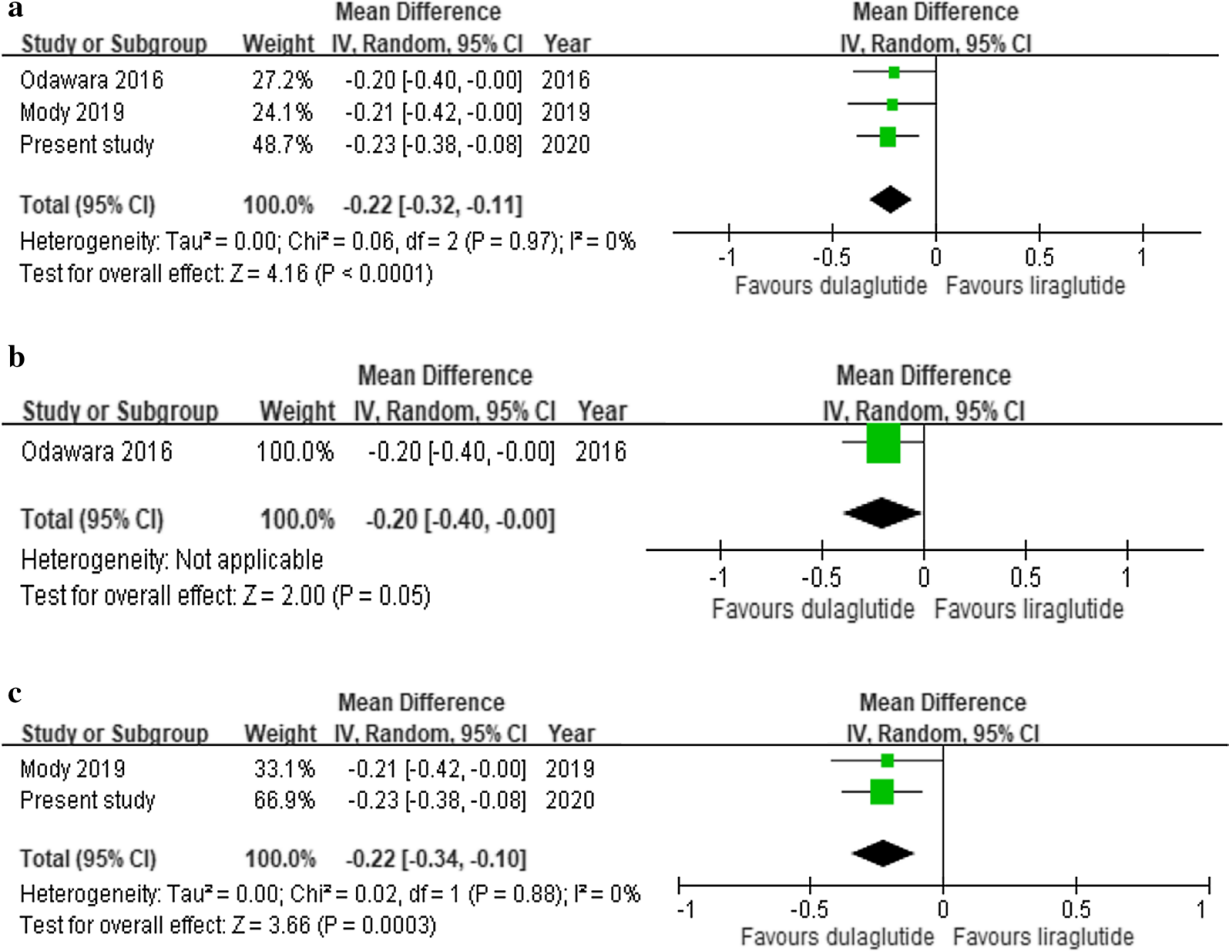
Forest plot of 12-month difference in HbA1c between dulaglutide and liraglutide. **a** All studies (including randomized controlled trials [RCTs] and observational studies), **b** RCTs only, and **c** observational studies only

Including the present study, we considered seven studies with up to a total of 5817 T2D patients for meta-analysis. Data extraction flow was detailed in Additional file 1: Appendix Figure S3. We excluded one study [14] with less than 6 months of follow-up and three studies [9, 13, 15] without sufficient outcome data. The characteristics of the included studies are provided in Additional file 1: Appendix Table S4. The use of dulaglutide versus liraglutide resulted in a significant HbA1c reduction; the pooled mean HbA1c difference between dulaglutide and liraglutide was − 0.17% (95% CI − 0.08 to − 0.26%) and − 0.22% (− 0.11 to − 0.32%) over the 6- and 12-month follow-ups, respectively (Additional file 1: Appendix Figure S4 and Fig. 3). However, there were comparable effects on weight loss and SBP reduction between two drugs (weight loss in Additional file 1: Appendix Figures S5 and S6, SBP reduction in Additional file 1: Appendix Figure S7 and S8). A considerable heterogeneity across the studies was noted for analyzing clinical effectiveness of treatments over a 6-month follow-up (*I*^2^ = 52% to 86%).

## Discussion

This large, real-world comparative effectiveness study of GLP-1RAs agents in a Taiwanese population with T2D across multiple medical institutions comprehensively evaluated clinical effectiveness of dulaglutide versus liraglutide. We found that dulaglutide versus liraglutide was associated with a greater HbA1c reduction at 12 months. The benefits of reducing body weight, blood pressure, and ALT levels were also found with these GLP-1RAs treatments, although between-group differences in these beneficial effects were not statistically significant. Moreover, across all pre-specified subgroups, there were consistent beneficial effects of dulaglutide versus liraglutide on HbA1c and SBP, some of which were of statistical significance. Treatment effects of dulaglutide versus liraglutide on clinical outcomes appeared to be modified by baseline HbA1c levels.

### Glycemic control

The AWARD-6 and Japanese trials first showed the non-inferiority of dulaglutide versus liraglutide at 26 weeks with a mean between-group difference in HbA1c change of − 0.06 to − 0.10% [5, 6]. The post hoc analysis of AWARD-6 showed the similar impact of dulaglutide and liraglutide on relative contribution of basal and postprandial hyperglycemia across HbA1c quartiles after 6 months of treatment [28]. With a longer follow-up period, the Japanese trial reported significant HbA1c reduction for dulaglutide versus liraglutide at 52 weeks with a mean between-group difference in HbA1c change of − 0.20% [7]. However, the small number of study participants in the Japanese trial [6, 7] and the selective and homogenous study populations in well-controlled trial settings [5–7] may limit the generalizability of study results to real-world diverse populations treated with GLP-1RAs. Our systematic review found that three real-world studies [10–12] have been conducted on the comparative effectiveness of dulaglutide versus liraglutide over a follow-up of 6 or 12 months in non-ethnically Chinese populations (Additional file 1: Appendix Table S4), showing a greater glycemic reduction with dulaglutide versus liraglutide [10, 12], except the Canadian study [11]. Compared to these previous studies with certain limitations such as having a more homogeneous population [5–7] or a limited number of patients (i.e., 417–1344 study subjects [5–7, 10–12]), this present large real-world study of GLP-1RAs in Taiwan reveals that the greater benefit of glycemic control was associated with the use of dulaglutide versus liraglutide over a follow-up of 3–12 months (Fig. 2). It is worth noting that the absolute glycemic reduction with dulaglutide and liraglutide found in this study is slightly smaller than that shown in previous trials. More frequent contact and monitoring, more intensive diabetes education, and better medication adherence in the well-controlled trial setting may contribute this discrepancy. Furthermore, patient populations differ among studies. For example, more complex comorbidities in our study population than previous trials’ populations [5–7] might have mitigated the glycemic effect of GLP-1RAs therapy. Nevertheless, consistent with previous studies [7, 10, 12], we found a significantly larger HbA1c reduction associated with dulaglutide versus liraglutide. Our meta-analysis that incorporated all existing data (including the present study) further demonstrated that the use of dulaglutide versus liraglutide possessed a superior glycemic control over a 6-month or 12-month follow-up (Fig. 3 and Additional file 1: Appendix Figure S4). The favorable glycemic control of dulaglutide over liraglutide might be explained by better adherence owing to its once-weekly regimen [12] and its long-lasting drug action attributable to the longer half-life [29].

### Body weight loss

Weight loss with individual GLP-1RAs drugs has been confirmed in T2D patients [30]. The AWARD-6 trial showed significantly larger body weight reduction associated with liraglutide versus dulaglutide (− 0.71 kg) at 26 weeks [5], but the Japanese trial showed no difference in weight loss between dulaglutide and liraglutide at 26 or 52 weeks [6, 7]. This implies that the weight loss effect of GLP-1RAs therapy may vary with patients’ baseline body weight and could be culture- or population-specific. Specifically, there was a significant weight reduction with GLP-1RAs use in the AWARD-6 trial participants, which predominantly comprised Caucasians with an average baseline weight of 94.1 kg [5], whereas there was no significant weight loss with GLP-1RAs use in the Japanese trial, which included Japanese patients with an average baseline body weight of 70.9 kg [6, 7]. Moreover, in Japanese populations, it has been reported that females with the treatment of dulaglutide or liraglutide generally had greater weight loss than males [31]. In our study of the ethnically Chinese population with an average baseline weight of 77.7 kg, the weight loss with dulaglutide (− 1.14 kg) or liraglutide (− 1.64 kg) was statistically significant. Although the weight loss of liraglutide compared to that of dulaglutide was greater, the between-treatment difference was not statistically significant. Another explanation for the weaker weight loss effect of GLP-1RAs in this study compared to that in the AWARD-6 trial [5] is the concomitant use of glucose-lowering agents (GLAs). Our study subjects were also treated with other GLAs which may have a weight gain effect (e.g., around 60% of patients on sulfonylurea which may have weight gain effect [32]), whereas the trial patients were treated with only metformin in addition to GLP-1RAs. The concomitant use of other GLAs with a potential weight gain effect may mask the weight benefit of GLP-1RAs. Nevertheless, the favorable weight benefit of liraglutide versus dulaglutide may be explained by the molecule size of the drug and the associated mechanism of weight loss. Because the dulaglutide molecule is larger than the liraglutide molecule, less dulaglutide is transported across the blood–brain barrier or through fenestrated capillaries and thus less effects for increasing satiety and inducing nausea in the central nervous system [29, 33]. As a result, dulaglutide may be less effective for weight reduction compared to liraglutide. Based on the meta-analysis for existing studies (including our study) (Additional file 1: Appendix Figures S5 and S6), the effect of weight loss for liraglutide versus dulaglutide was greater over a 6-month follow-up but comparable over a 12-month follow-up.

### Blood pressure control

Reduced blood pressure with GLP-1RAs treatment has been documented in recent cardiovascular outcome trials [34, 35] and linked to better cardiovascular outcomes in GLP-1RAs users [36]. In previous head-to-head comparison trials of GLP-1RAs [5–7], blood pressure reduction was found in both dulaglutide and liraglutide groups, while the between-group difference in blood pressure change was not statistically significant. In the present study, only the use of dulaglutide was associated with a significant blood pressure reduction, which is consistent with a recent real-world study from Italy [10]. This discrepancy between trials [5–7] and real-world studies (our study and the Italian study [10]) may be explained by more complicated comorbidities and more use of antihypertension agents in the real-world patient populations, which might mask the blood pressure reduction effects of GLP-1RAs therapy. Limited evidence for blood pressure control between dulaglutide and liraglutide (Additional file 1: Appendix Figures S7 and S8) suggests a need for future research.

### Liver function

Previous studies have supported a reduced ALT level associated with liraglutide use compared to either placebo [37] or no GLAs use [38]. Consistent with these findings, the present study found that both dulaglutide and liraglutide were associated with a significant ALT decline at 12 months, despite no significant difference in the ALT decline between two treatments. In addition, we found that the magnitude of ALT reduction with GLP-1RAs use was greater in the subgroup of patients with abnormal liver function, which was indicated with a significant interaction between treatment groups (dulaglutide versus liraglutide) and ALT levels (> UNL versus ≤ UNL) (Additional file 1: Appendix Figure S2d). Future research is warranted to corroborate this finding to determine whether the liver outcomes associated with GLP-1RAs use vary with individual GLP-1RAs drugs or patients’ underlying liver function.

### Renal function

Although previous clinical studies have revealed either no change in eGFR (i.e., liraglutide versus placebo [39]) or reduced eGFR declines (eGFR preservation) (i.e., dulaglutide versus insulin or placebo [35, 40] or liraglutide versus placebo [41]), there has been no head-to-head comparison of dulaglutide versus and liraglutide on renal outcomes. The present study found that a slight decrease in eGFR was associated with both GLP-1RAs treatments. However, our subgroup analyses further showed that among patients with chronic renal impairment (eGFR < 60 mL/min/1.73 m^2^), a slight increase in eGFR was associated with both GLP-1RAs treatments, and that the interaction between treatment groups (dulaglutide versus liraglutide) and eGFR levels (eGFR ≥ 60 versus < 60 mL/min/1.73 m^2^) was statistically significant (Additional file 1: Appendix Figure S2e). This study adds supporting evidence of favorable renal effects with GLP-1RAs therapy in a real-world T2D population that had a relatively poor renal function. Future research should be conducted to determine whether the renal benefit of different GLP-1RAs varies by patients’ underlying renal function.

The present study has several strengths compared to previous studies. First, our study cohort was derived from large EMRs across multiple institutions at different levels of hospitals throughout Taiwan that comprised a diverse real-world T2D population along with their individual-level detailed laboratory measurements, which are typically lacking in administrative datasets, to enrich our analyses. Second, this is the largest real-world Asian study on the comparative effectiveness of GLP-1RAs drugs among a T2D population. Third, we considered a wide range of biochemical markers in the analyses. We are thus able to either corroborate the findings from existing studies or provide additional evidence of clinical effects of GLP-1RAs. Lastly, our rigorous analytic procedures with a series of sensitivity and subgroup analyses that varied with different clinical scenarios ensure the robustness of our study findings and their generalizability to diverse real-world T2D populations treated with GLP-1RAs.

This study also has several limitations. First, as a retrospective design study, possible confounding by indication and unmeasured confounding might not have been avoided. However, we implemented the rigorous PS procedures (e.g., matching, weighting) to enhance between-group comparability and the sensitivity analyses to account for patients’ medication use behaviors (i.e., as-treated scenario, stable GLP-1RAs users) and possible healthy user bias to minimize potential confounding and bias commonly seen in retrospective studies. Second, the present study only assessed the clinical effectiveness of GLP-1RAs in terms of short-term clinical biochemical marker changes (e.g., HbA1c, eGFR), while the hard endpoints of treatments (e.g., cardiovascular disease, death, progression to end-stage renal disease) were not measured. To date, there is a lack of direct head-to-head comparative trials of GLP-1RAs on the long-term cardiovascular safety and mortality, but there are indirect comparisons from three network meta-analysis studies [42–44]. This suggests that future studies with the long-term follow-up period on hard outcomes among GLP-1RAs are needed. Third, comparison of dulaglutide with semaglutide would be also important as they are both once-weekly injections. However, semaglutide was not available in Taiwan during our study period and thus was not analyzed in this study. Future research for comparative effectiveness of dulaglutide versus semaglutide is needed. Lastly, it is possible that our study patients had visited other medical institutions outside the CGRD system. The lack of continuity in medical visits would affect the completeness of our patient records. However, because our primary analysis was based on study patients with at least one HbA1c value in the year of the follow-up and our sensitivity analysis was also performed by assessing stable GLP-1RAs users, our study patients are most likely to be loyal patients in the CGRD system. We further analyzed the rates of loss to follow-up between treatment groups and found similar rates between two treatment groups (dulaglutide versus liraglutide: 12.9% versus 14.3%). This implies that the potential bias attributable to incomplete follow-up records could be negligible.

## Conclusion

In this large real-world T2D population treated with GLP-1RAs, dulaglutide was associated with more favorable glycemic control compared to liraglutide. Weight loss, blood pressure reduction, and improved liver and renal functions after GLP-1RAs treatments were also found. The differences in these clinical outcomes between dulaglutide and liraglutide were comparable. Future research on the comparative effectiveness of among other GLP-1RAs drugs and a comparison of GLP-1RAs with other GLAs is warranted to support the clinical rationale of selecting GLP-1RAs treatment.

## Supplementary information

**Additional file1 : Figure S1.** Distribution of kernel density of the propensity score distribution in dulaglutide and liraglutide users before and after propensity score matching. **Table S1.** Search strategy and key terms for meta-analysis. **Table S2.** Comparison of clinical effectiveness between liraglutide and dulaglutide (sensitivity analyses). **Table S3. **Subgroup analyses for comparison of clinical effectiveness changes between dulaglutide and liraglutide at 12 months (based on the propensity-score-matched sample). **Figure S2.** Changes in clinical effectiveness between dulaglutide and liraglutide at 12 months stratified by patient subgroup (based on the propensity-score-matched sample). **Figure S3.** Flow chart of selection of studies included in the meta-analysis. **Table S4.** Summary of existing studies that head-to-head compared dulaglutide and liraglutide. **Figure S4.** Forest plot of 6-month difference in HbA1c between dulaglutide and liraglutide. **Figure S5.** Forest plot of 12-month difference in weight loss between dulaglutide and liraglutide. **Figure S6.** Forest plot of 6-month difference in weight loss between dulaglutide and liraglutide. **Figure S7.** Forest plot of 12-month difference in systolic blood pressure change between dulaglutide and liraglutide. **Figure S8.** Forest plot of 6-month difference in systolic blood pressure change between dulaglutide and liraglutide.

## Data Availability

Data sharing is not applicable to this study as data management and analysis were performed on a statistics server through remote access in Chang Gung Medical Foundation in Taiwan, for privacy and safety concerns.

## Abbreviations

GLP-1RAs: Glucagon-like peptide-1 receptor agonists
T2D: Type 2 diabetes
EMRs: Electronic medical records
CGRD: Chang Gung Research Database
ICD-9: International Classification of Diseases, Ninth Revision
ICD-10: International Classification of Diseases, Tenth Revision
SBP: Systolic blood pressure
ALT: Alanine aminotransferase
eGFR: Estimated glomerular filtration rate
PS: Propensity score
ITT: Intention-to-treat
DPP-4i: Dipeptidyl peptidase 4 inhibitor
SGLT-2i: Sodium glucose cotransporter 2 inhibitor
IPTW: Inverse probability of treatment weighting
SMRW: Standardized mortality ratio weighting
