# Supplementary material for: Comparative effectiveness of dulaglutide versus liraglutide in Asian type 2 diabetes patients: a multi-institutional cohort study and meta-analysis
Source: Cardiovasc Diabetol. 2020 Oct 9;19:172. doi: 10.1186/s12933-020-01148-8 (PMC7547475; doi:10.1186/s12933-020-01148-8)
Supplement: Supplementary file 1 — Additional file1 : Figure S1. Distribution of kernel density of the propensity score distribution in dulaglutide and liraglutide users before and after propensity score matching. Table S1. Search strategy and key terms for meta-analysis. Table S2. Comparison of clinical effectiveness between liraglutide and dulaglutide (sensitivity analyses). Table S3. Subgroup analyses for comparison of clinical effectiveness changes between dulaglutide and liraglutide at 12 months (based on the propensity-score-matched sample). Figure S2. Changes in clinical effectiveness between dulaglutide and liraglutide at 12 months stratified by patient subgroup (based on the propensity-score-matched sample). Figure S3. Flow chart of selection of studies included in the meta-analysis. Table S4. Summary of existing studies that head-to-head compared dulaglutide and liraglutide. Figure S4. Forest plot of 6-month difference in HbA1c between dulaglutide and liraglutide. Figure S5. Forest plot of 12-month difference in weight loss between dulaglutide and liraglutide. Figure S6. Forest plot of 6-month difference in weight loss between dulaglutide and liraglutide. Figure S7. Forest plot of 12-month difference in systolic blood pressure change between dulaglutide and liraglutide. Figure S8. Forest plot of 6-month difference in systolic blood pressure change between dulaglutide and liraglutide. [file 12933_2020_1148_MOESM1_ESM.docx]

Appendix Figure S1. Distribution of kernel density of the propensity score distribution in dulaglutide and liraglutide users before and after propensity score matching


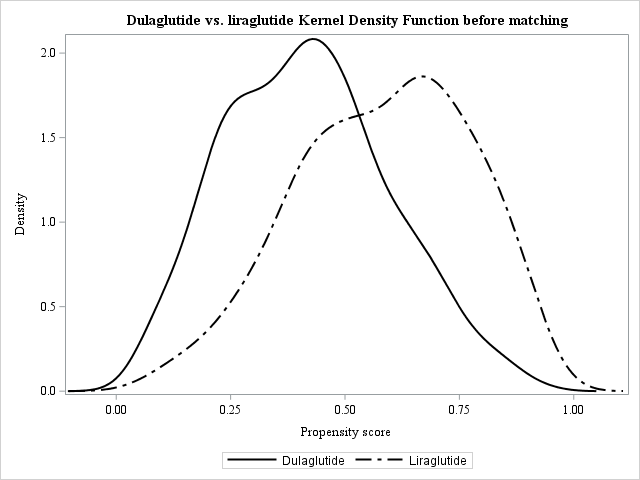


(b)


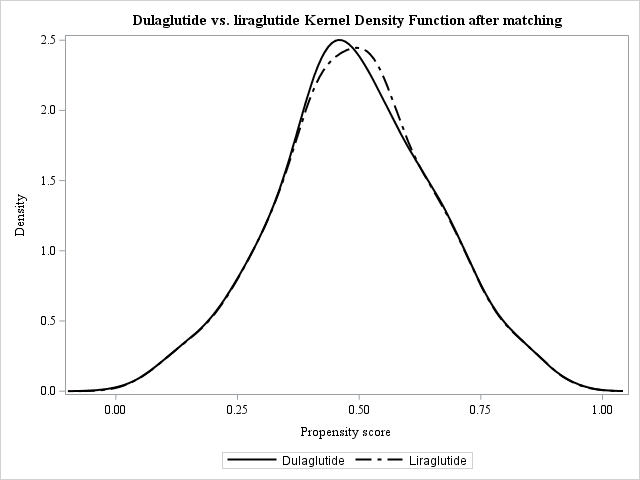


Legend: (a) Before propensity score matching, and (b) after propensity score matching.

Appendix Table S1. Search strategy and key terms for meta-analysis

| # | Search |
| --- | --- |
|  | **Patient** |
| 1 | Type 2 diabetes mellitus |
|  | **Intervention and comparison** |
| 2 | Dulaglutide |
| 3 | Liraglutide |
|  | **Outcome** |
| 4 | Glycated hemoglobin |
| 5 | HbA1c |
| 6 | 4 or 5 |
| 7 | Body weight |
| 8 | Body mass index |
| 9 | 7 or 8 |
| 10 | Blood pressure |
| 11 | Renal |
| 12 | Glomerular filtration rate |
| 13 | 11 or 12 |
| 14 | Liver |
| 15 | Alanine aminotransferase |
| 16 | 14 or 15 |
| 17 | 6 or 9 or 10 or 13 or 16 |
| 18 | 1 and 2 and 3 and 17 |

Appendix Table S2. Comparison of clinical effectiveness between liraglutide and dulaglutide (sensitivity analyses)

|  | Dulaglutide | | Liraglutide | | Dulaglutide versus liraglutide |
| --- | --- | --- | --- | --- | --- |
|  | Baseline (SD) | Change from baseline (SE)^1^ | Baseline (SD) | Change from baseline (SE)^1^ | Mean difference (95% CI)^2^ |
| **Sensitivity 1: as-treated scenario** | |  |  |  |  |
| HbA1c (%) | 9.36 (1.66) | -1.07 (0.05)*** | 9.33 (1.57) | -0.77 (0.05)*** | -0.30 (-0.45 to -0.16)*** |
| Weight (kg) | 77.83 (17.69) | -1.06 (0.27)*** | 77.53 (16.81) | -1.68 (0.26)*** | 0.61 (-0.11 to 1.35) |
| SBP (mmHg) | 140.71 (19.94) | -1.97 (0.67)** | 140.24 (20.24) | -0.49 (0.70) | -1.47 (-3.39 to 0.43) |
| ALT (U/L) | 36.10 (30.23) | -2.82 (0.93)** | 35.47 (32.66) | -3.19 (0.96)*** | 0.36 (-2.27 to 2.99) |
| eGFR (ml/min/1.73 m^2^) | 82.43 (38.21) | -2.71 (0.57)*** | 82.13 (35.69) | -1.95 (0.55)*** | -0.76 (-2.33 to 0.81) |
| **Sensitivity 2: only stable users of GLP-1ra** | | |  |  |  |
| HbA1c (%) | 9.29 (1.62) | -1.03 (0.06)*** | 9.31 (1.57) | -0.84 (0.07)*** | -0.19 (-0.38 to -0.01)* |
| Weight (kg) | 78.92 (18.08) | -1.50 (0.37)*** | 78.83 (16.50) | -2.27 (0.37)*** | 0.76 (-0.27 to 1.80) |
| SBP (mmHg) | 140.53 (19.60) | 2.38 (0.82)** | 139.90 (20.16) | -0.58 (0.91) | -1.79 (-4.21 to 0.61) |
| ALT (U/L) | 34.78 (28.31) | -2.03 (0.94)* | 36.18 (32.08) | -3.56 (1.12)** | 1.52 (-1.33 to 4.37) |
| eGFR (ml/min/1.73 m^2^) | 81.45 (37.91) | -1.24 (0.78) | 81.18 (33.51) | -1.64 (0.74)* | 0.40 (-1.74 to 2.54) |
| **Sensitivity 3: healthy user bias adjustment (excluding GLP-1ra users also treated with DPP-4i or SGLT-2i)** | | | | |  |
| HbA1c (%) | 9.34 (1.67) | -1.06 (0.05)*** | 9.33 (1.59) | -0.82 (0.05)*** | -0.24 (-0.39 to -0.08)** |
| Weight (kg) | 77.82 (17.64) | -1.19 (0.32)*** | 77.22 (16.73) | -1.61 (0.32)*** | 0.41 (-0.48 to 1.31) |
| SBP (mmHg) | 141.02 (19.76) | -2.50 (0.70)*** | 140.27 (20.43) | -0.49 (0.75) | -2.00 (-4.04 to 0.02) |
| ALT (U/L) | 36.41 (30.36) | -3.37 (0.87)*** | 35.24 (32.68) | -3.72 (0.97)*** | 0.34 (-2.21 to 2.90) |
| eGFR (ml/min/1.73 m^2^) | 83.20 (38.27) | -2.03 (0.73)** | 82.11 (35.64) | -2.04 (0.65)** | 0.01 (-1.90 to 1.93) |
| **Sensitivity 4: IPTW-based sample** | | | | | |
| HbA1c (%) | 9.35 (2.37) | -1.06 (0.04)*** | 9.38 (2.26) | -0.86 (0.04)*** | -0.20 (-0.33 to -0.07)** |
| Weight (kg) | 77.71 (25.11) | -1.27 (0.27)*** | 77.44 (23.62) | -1.62 (0.27)*** | 0.35 (-0.40 to 1.10) |
| SBP (mmHg) | 139.80 (27.98) | -1.53 (0.58)** | 139.88 (28.11) | -1.13 (0.60) | -0.40 (-2.05 to 1.24) |
| ALT (U/L) | 35.38 (42.42) | -2.30 (0.70)** | 35.26 (45.30) | -2.77 (0.82)*** | 0.46 (-1.64 to 2.58) |
| eGFR (ml/min/1.73 m^2^) | 82.16 (53.94) | -2.45 (0.57)*** | 82.47 (51.24) | -2.71 (0.54)*** | 0.25 (-1.29 to 1.80) |
| **Sensitivity 5: SMRW-based sample** | | | | | |
| HbA1c (%) | 9.42 (1.75) | -1.11 (0.04)*** | 9.46 (1.66) | -0.90 (0.04)*** | -0.20 (-0.33 to -0.07)** |
| Weight (kg) | 77.93 (17.78) | -1.28 (0.27)*** | 77.13 (16.52) | -1.39 (0.26)*** | 0.11 (-0.63 to 0.86) |
| SBP (mmHg) | 139.72 (19.77) | -1.57 (0.59)** | 139.67 (19.97) | -0.78 (0.60) | -0.79 (-2.46 to 0.88) |
| ALT (U/L) | 35.56 (31.49) | -2.58 (0.72)*** | 34.86 (31.52) | -2.68 (0.81)*** | 0.09 (-2.04 to 2.23) |
| eGFR (ml/min/1.73 m^2^) | 82.04 (39.17) | -2.57 (0.58)*** | 81.60 (37.36) | -2.82 (0.53)*** | 0.24 (-1.31 to 1.80) |

Abbreviations: SD, standard deviation; CI, confidence interval; SBP, systolic blood pressure; eGFR, estimated glomerular filtration rate; ALT, alanine aminotransferase; GLP-1ra, glucagon-like peptide-1 receptor agonist; DPP-4i, dipeptidyl peptidase-4 inhibitor; SGLT-2i, sodium-glucose transport protein-2 inhibitor; IPTW, inverse probability of treatment weighting; SMRW, standardized mortality ratio weighting.

Notes:

1. “Change from baseline” in each treatment group was tested using the paired *t*-test.
2. “Mean difference” between treatment groups was tested using the two-sample *t*-test.

*, **, and *** refer to *p*-value < 0.05, < 0.01, and < 0.001, respectively.

Appendix Table S3. Subgroup analyses for comparison of clinical effectiveness changes between dulaglutide and liraglutide at 12 months (based on the propensity-score-matched sample)

|  | Dulaglutide | | Liraglutide | | Dulaglutide vs. liraglutide |
| --- | --- | --- | --- | --- | --- |
|  | Baseline  (SD) | Change from baseline  (SE)^1^ | Baseline  (SD) | Change from baseline  (SE)^1^ | Mean difference  (95% CI)^2^ |
| **HbA1c ≥ 9% (n = 1,161)** | | | | | |
| HbA1c (%) | 10.41 (1.28) | -1.63 (0.07)*** | 10.32 (1.15) | -1.35 (0.07)*** | -0.28 (-0.48 to -0.08)** |
| Weight (kg) | 76.10 (16.98) | -0.39 (0.38) | 75.27 (15.94) | -0.49 (0.36) | 0.10 (-0.94 to 1.14) |
| SBP (mmHg) | 141.39 (20.05) | -2.33 (0.91)* | 139.82 (20.21) | 0.82 (0.95) | -3.15 (-5.75 to -0.55)* |
| ALT (U/L) | 36.29 (31.24) | -2.94 (1.10)** | 33.78 (28.04) | -3.54 (0.95) | 0.59 (-2.25 to 3.44) |
| eGFR (ml/min/1.73 m^2^) | 82.64 (39.29) | -2.33 (0.92)* | 81.20 (36.70) | -4.13 (0.82) | 1.79 (-0.63 to 4.22) |
| **HbA1c < 9% (n = 805)** | | | | | |
| HbA1c (%) | 7.89 (0.78) | -0.26 (0.05)*** | 7.88 (0.76) | -0.07 (0.06) | -0.19 (-0.36 to -0.02)* |
| Weight (kg) | 80.27 (18.40) | -2.21 (0.49)*** | 80.87 (17.51) | -3.35 (0.54)*** | 1.13 (-0.30 to 2.57) |
| SBP (mmHg) | 139.76 (19.76) | -2.66 (1.06)* | 140.87 (20.29) | -2.60 (1.10)* | -0.05 (-3.06 to 2.94) |
| ALT (U/L) | 35.84 (28.77) | -3.27 (1.23)** | 37.97 (38.37) | -3.82 (1.78)* | 0.54 (-3.69 to 4.78) |
| eGFR (ml/min/1.73 m^2^) | 82.13 (36.68) | -1.73 (1.03) | 83.44 (34.14) | 0.33 (0.95) | -2.06 (-4.83 to 0.70) |
| **Age ≥65 years (n = 621)** | | | | | |
| HbA1c (%) | 9.42 (1.59) | -1.30 (0.09)*** | 9.40 (1.47) | -0.94 (0.09)*** | -0.36 (-0.62 to -0.10)** |
| Weight (kg) | 69.46 (13.10) | -0.58 (0.43) | 70.71 (12.07) | -1.89 (0.40)*** | 1.30 (0.13 to 2.48)* |
| SBP (mmHg) | 141.42 (19.94) | -2.78 (1.27)* | 141.71 (20.35) | 0.53 (1.47) | -3.32 (-7.14 to 0.49) |
| ALT (U/L) | 29.01 (23.11) | -1.47 (1.17) | 27.66 (29.84) | -2.72 (1.69) | 1.24 (-2.77 to 5.27) |
| eGFR (ml/min/1.73 m^2^) | 61.78 (28.20) | -1.26 (0.99) | 61.23 (27.43) | -1.97 (0.89)* | 0.71 (-1.92 to 3.34) |
| **Age <65 years (n = 1,345)** | | | | | |
| HbA1c (%) | 9.34 (1.69) | -0.95 (0.06)*** | 9.30 (1.61) | -0.78 (0.06)*** | -0.16 (-0.34 to 0.01) |
| Weight (kg) | 81.80 (18.21) | -1.41 (0.40)*** | 80.60 (17.72) | -1.53 (0.41) | 0.12 (-1.00 to 1.25) |
| SBP (mmHg) | 140.38 (19.94) | -2.32 (0.82)** | 139.59 (20.17) | -1.05 (0.81) | -1.26 (-3.54 to 1.01) |
| ALT (U/L) | 39.46 (32.55) | -3.84 (1.07)*** | 38.98 (33.27) | -4.07 (1.08)*** | 0.22 (-2.77 to 3.23) |
| eGFR (ml/min/1.73 m^2^) | 92.21 (38.45) | -2.47 (0.90)** | 91.53 (35.00) | -2.48 (0.81)** | 0.01 (-2.37 to 2.40) |
| **eGFR ≥ 60 ml/min/1.73 m^2^ (n = 1,416)** | | | | | |
| HbA1c (%) | 9.31 (1.65) | -1.00 (0.06)*** | 9.29 (1.54) | -0.77 (0.06)*** | -0.23 (-0.40 to -0.06)** |
| Weight (kg) | 78.74 (18.21) | -1.31 (0.38)*** | 78.52 (17.29) | -1.76 (0.38)*** | 0.45 (-0.60 to 1.50) |
| SBP (mmHg) | 139.79 (19.29) | -1.46 (0.77) | 139.61 (19.64) | -1.40 (0.79) | -0.05 (-2.24 to 2.13) |
| ALT (U/L) | 40.04 (32.83) | -4.67 (1.06)*** | 38.93 (36.02) | -4.74 (1.20)*** | 0.06 (-3.08 to 3.22) |
| eGFR (ml/min/1.73 m^2^) | 99.59 (29.66) | -5.00 (0.84)*** | 98.30 (27.36) | -4.59 (0.78)*** | -0.41 (-2.67 to 1.84) |
| **eGFR < 60 ml/min/1.73 m^2^ (n = 550)** | | | | | |
| HbA1c (%) | 9.49 (1.68) | -1.22 (0.10)*** | 9.44 (1.62) | -1.00 (0.10)*** | -0.22 (-0.51 to 0.06) |
| Weight (kg) | 75.58 (16.11) | -0.71 (0.48) | 74.99 (15.23) | -1.33 (0.52)** | 0.62 (-0.78 to 2.03) |
| SBP (mmHg) | 143.09 (21.37) | -5.06 (1.44)*** | 141.87 (21.67) | 1.60 (1.57) | -6.67 (-10.87 to -2.47)** |
| ALT (U/L) | 25.97 (18.73) | 1.01 (1.01) | 26.56 (19.06) | -0.84 (1.05) | 1.86 (-1.00 to 4.73) |
| eGFR (ml/min/1.73 m^2^) | 38.24 (15.54) | 5.43 (1.04)*** | 40.50 (14.83) | 3.49 (0.88)*** | 1.94 (-0.75 to 4.64) |
| **ALT > UNL (n = 632)** | | | | | |
| HbA1c (%) | 9.25 (1.56) | -1.01 (0.08)*** | 9.28 (1.54) | -0.99 (0.10)*** | -0.02 (-0.28 to 0.23) |
| Weight (kg) | 84.75 (19.28) | -2.44 (0.60)*** | 82.93 (18.85) | -2.36 (0.61)*** | -0.08 (-1.77 to 1.61) |
| SBP (mmHg) | 141.81 (18.60) | -3.10 (1.18)** | 141.06 (19.13) | -1.01 (1.24) | -2.09 (-5.47 to 1.28) |
| ALT (U/L) | 65.07 (36.98) | -19.13 (2.00)*** | 67.79 (42.57) | -22.79 (2.40)*** | 3.65 (-2.45 to 9.76) |
| eGFR (ml/min/1.73 m^2^) | 92.08 (31.85) | -2.88 (1.22)* | 91.38 (32.71) | -1.81 (1.06) | -1.07 (-4.28 to 2.14) |
| **ALT ≤ UNL (n = 1,334)** | | | | | |
| HbA1c (%) | 9.42 (1.71) | -1.09 (0.06)*** | 9.35 (1.58) | -0.76 (0.06)*** | -0.32 (-0.51 to -0.14)*** |
| Weight (kg) | 74.37 (15.76) | -0.50 (0.34) | 75.11 (15.21) | -1.32 (0.35)*** | 0.82 (-0.14 to 1.80) |
| SBP (mmHg) | 140.16 (20.57) | -2.15 (0.85)* | 139.88 (20.72) | -0.36 (0.89) | -1.78 (-4.21 to 0.63) |
| ALT (U/L) | 21.60 (7.52) | 4.95 (0.47)*** | 21.00 (7.54) | 4.19 (0.51)*** | 0.04 (-1.32 to 1.41) |
| eGFR (ml/min/1.73 m^2^) | 77.59 (40.18) | -1.68 (0.83)* | 77.99 (36.21) | -2.56 (0.77)** | 0.87 (-1.35 to 3.11) |
| **BMI ≥ 27 kg/m^2^ (n = 1,197)** | | | | | |
| HbA1c (%) | 9.22 (1.63) | -0.95 (0.06)*** | 9.13 (1.55) | -0.70 (0.06)*** | -0.24 (-0.43 to -0.05)** |
| Weight (kg) | 86.80 (16.11) | -3.23 (0.41)*** | 85.67 (15.11) | -3.64 (0.40)*** | 0.40 (-0.73 to 1.54) |
| SBP (mmHg) | 142.80 (19.13) | -2.67 (0.90)** | 142.44 (19.14) | -1.46 (0.89) | -1.21 (-3.70 to 1.28) |
| ALT (U/L) | 40.40 (33.50) | -4.66 (1.14)*** | 38.46 (33.23) | -3.85 (1.13)*** | -0.81 (-3.97 to 2.34) |
| eGFR (ml/min/1.73 m^2^) | 83.24 (36.42) | -1.34 (0.90) | 82.51 (34.62) | -1.67 (0.79)* | 0.32 (-2.04 to 2.69) |
| **BMI < 27 kg/m^2^ (n = 769)** | | | | | |
| HbA1c (%) | 9.58 (1.68) | -1.24 (0.09)*** | 9.65 (1.54) | -1.03 (0.08) | -0.20 (-0.45 to 0.03) |
| Weight (kg) | 63.85 (8.72) | 2.11 (0.40)*** | 64.89 (10.21) | 1.45 (0.43)*** | 0.66 (-0.49 to 1.82) |
| SBP (mmHg) | 137.46 (20.75) | -2.15 (1.08)* | 136.84 (21.42) | 0.82 (1.21) | -2.98 (-6.18 to 0.22) |
| ALT (U/L) | 29.40 (22.72) | -0.61 (1.10) | 30.83 (31.23) | -3.35 (1.54)* | 2.73 (-0.99 to 6.47) |
| eGFR (ml/min/1.73 m^2^) | 81.16 (40.85) | -3.24 (1.06)** | 81.53 (37.33) | -3.35 (1.01)** | 0.11 (-2.78 to 3.00) |

Abbreviations: SD, standard deviation; CI, confidence interval; SBP, systolic blood pressure; eGFR, estimated glomerular filtration rate; ALT, alanine aminotransferase; UNL, upper normal limit; BMI, body mass index.

Notes:

1. “Change from baseline” in each treatment group was tested using the paired *t*-test.
2. “Mean difference” between treatment groups was tested using the two-sample *t*-test.

*, **, and *** refer to *p*-value < 0.05, < 0.01, and < 0.001, respectively.

Appendix Figure S2. Changes in clinical effectiveness between dulaglutide and liraglutide at 12 months stratified by patient subgroup (based on the propensity-score-matched sample)

**
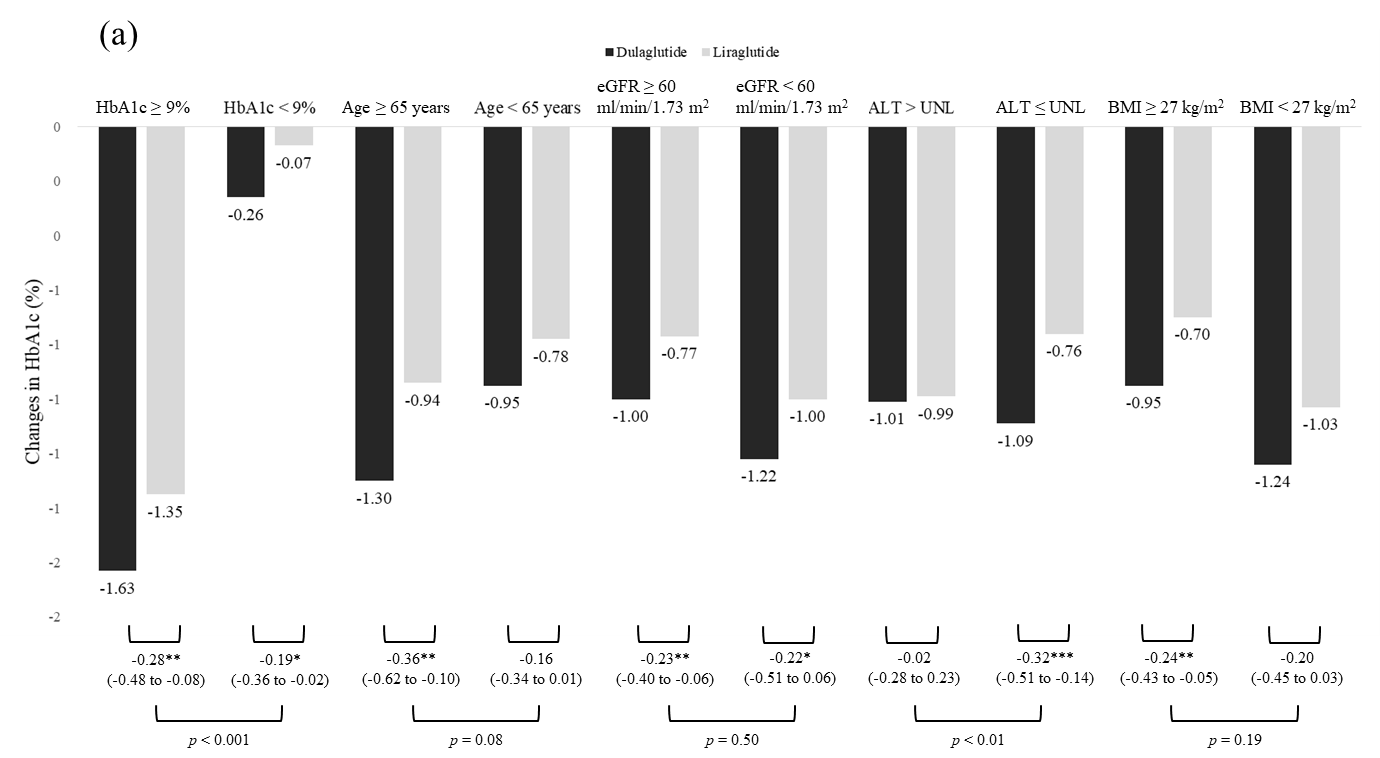
**

**
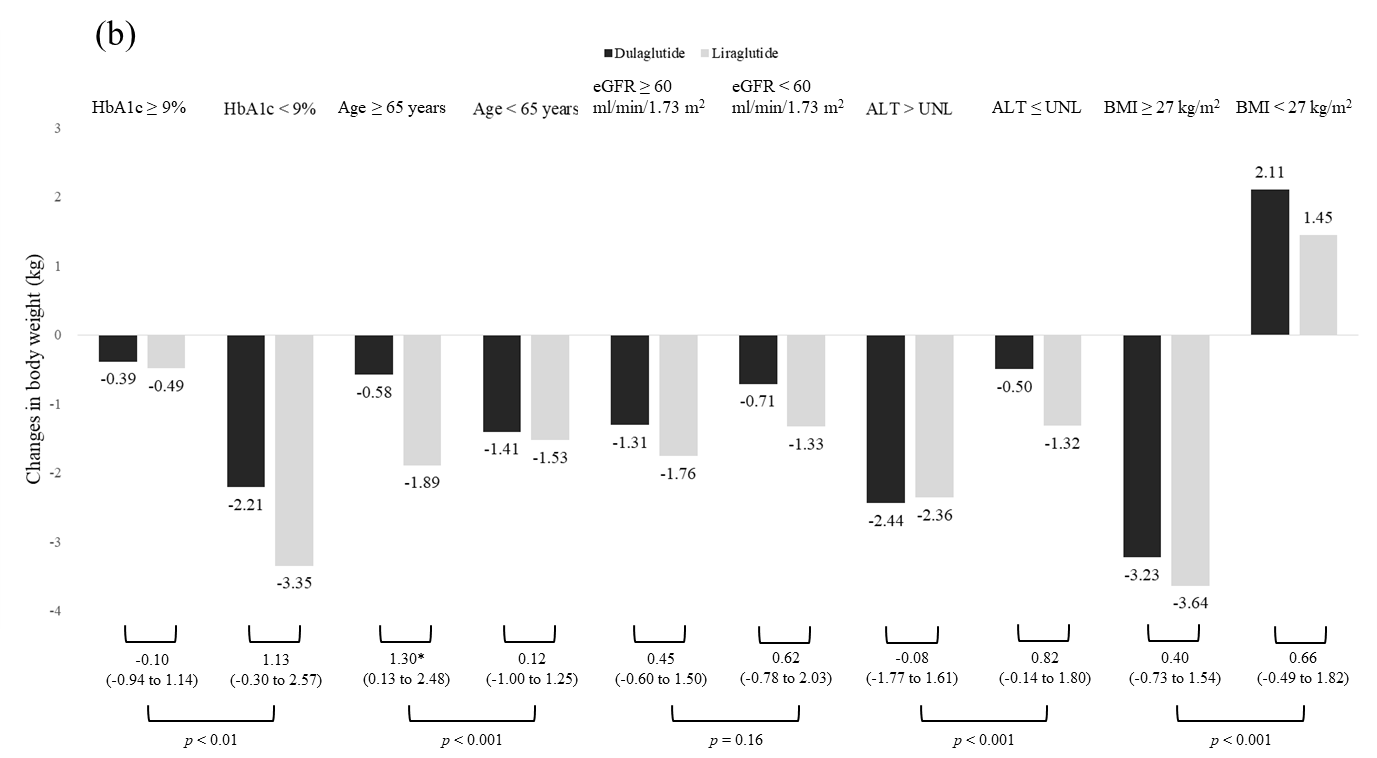
**

**
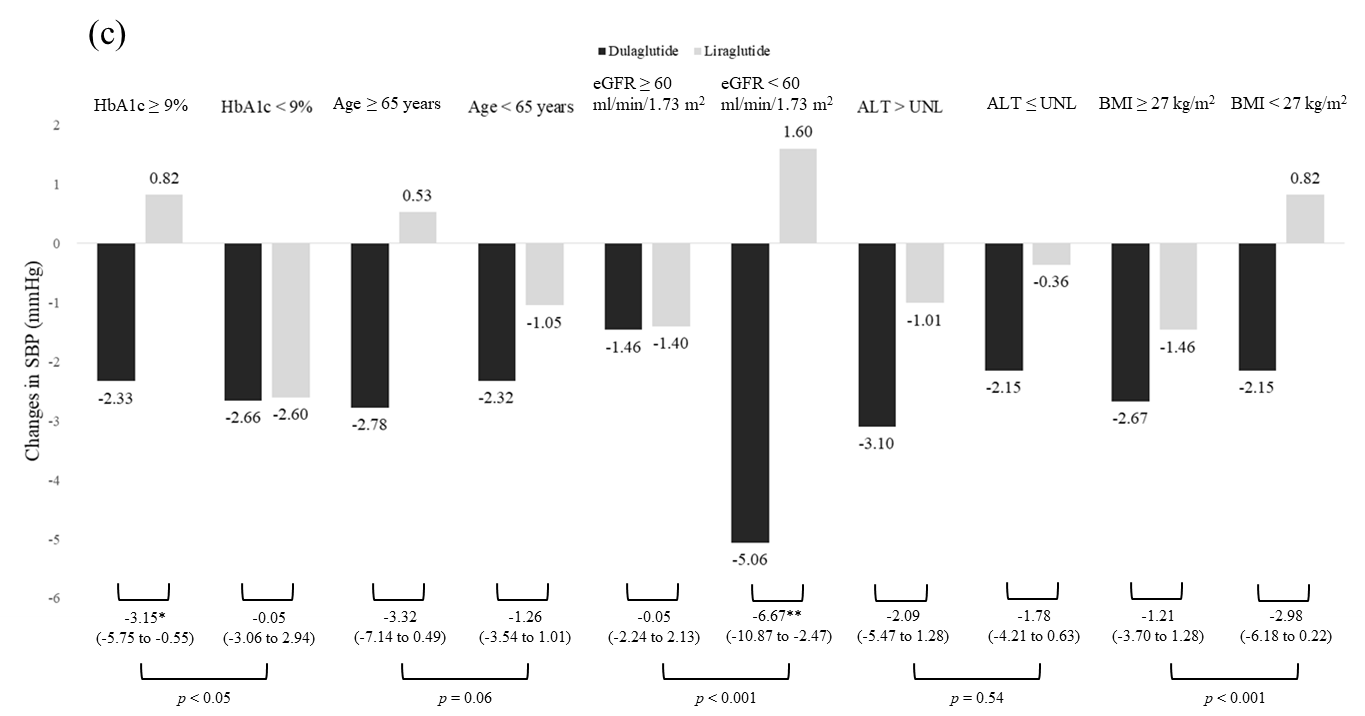
**

**
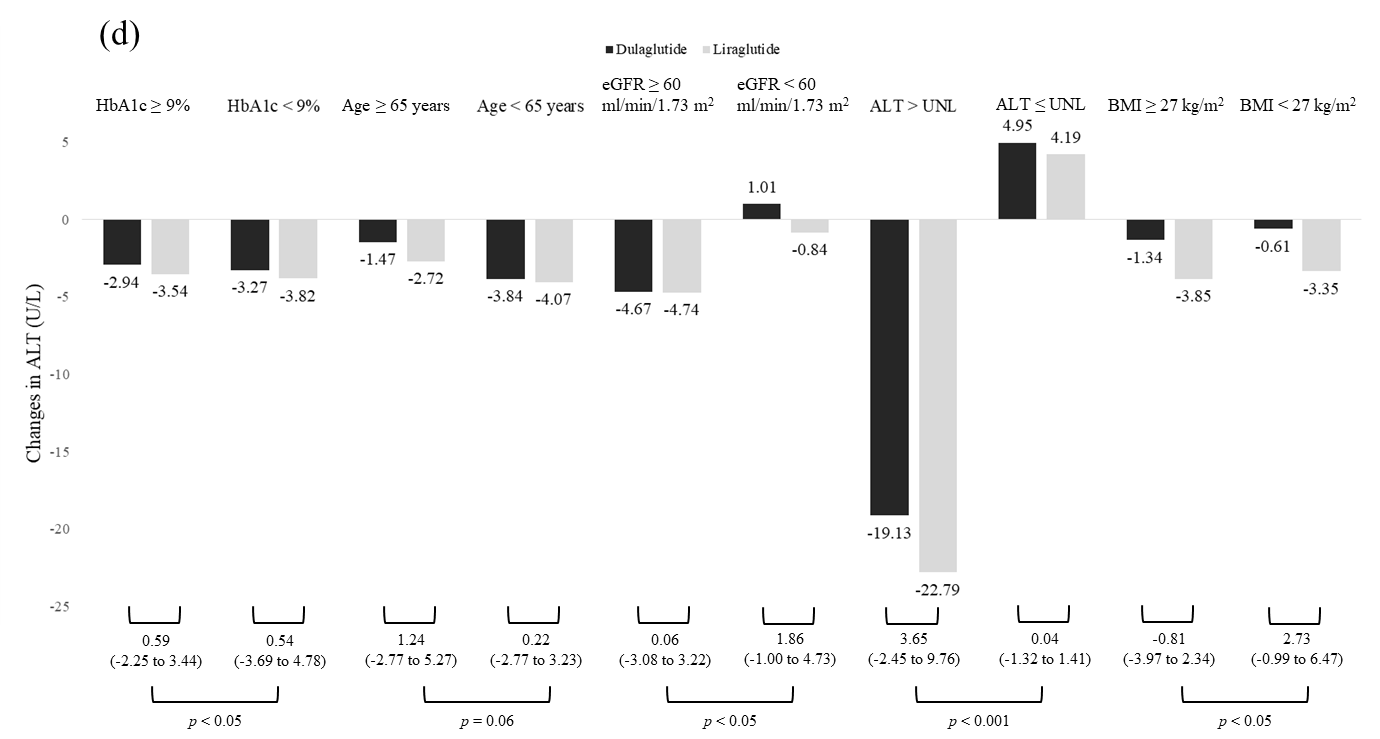
**

**
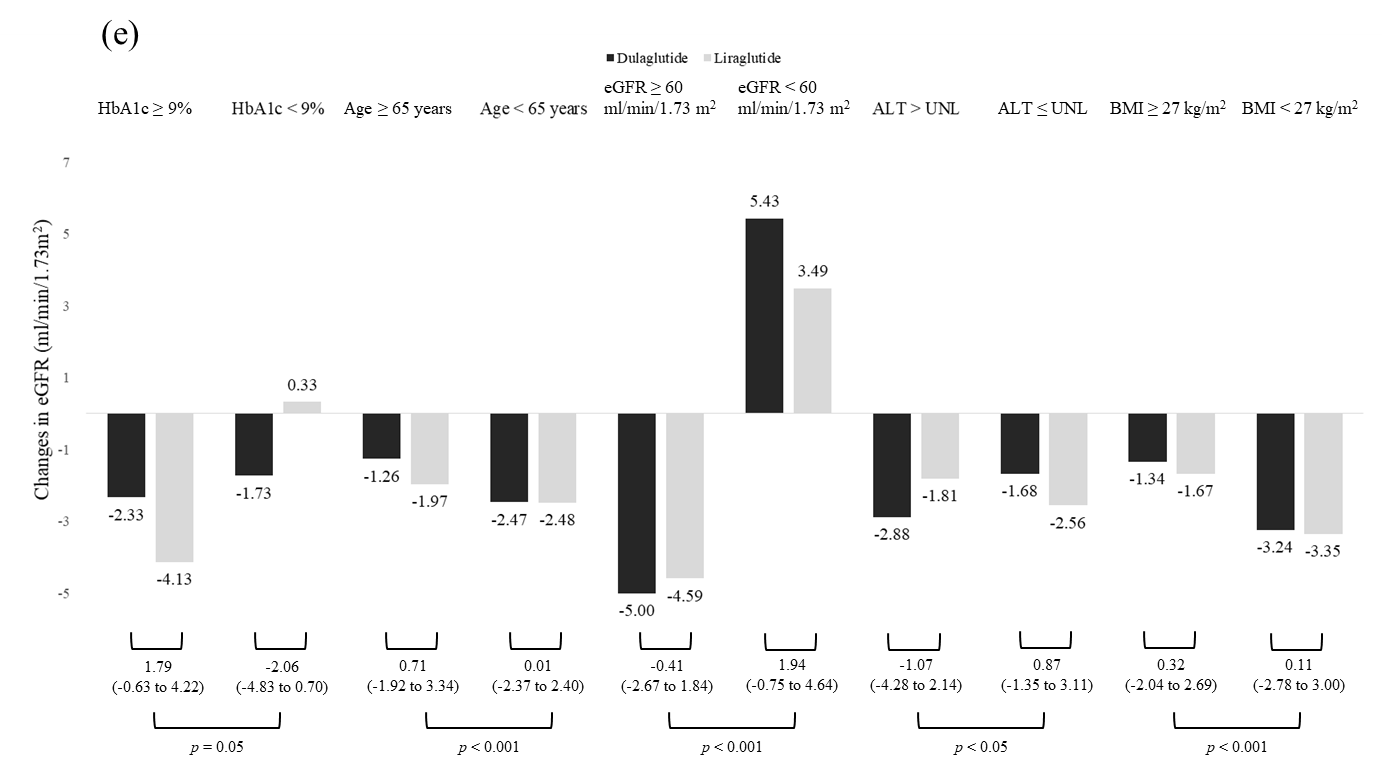
**

Legend: (a) Change in HbA1c from baseline to month 12 in different subgroups, (b) change in body weight from baseline to month 12 in different subgroups, (c) change in systolic blood pressure (SBP) from baseline to month 12 in different subgroups, (d) change in alanine aminotransferase (ALT) from baseline to month 12 in different subgroups, and (e) change in estimated glomerular filtration rate (eGFR) from baseline to month 12 in different subgroups.

Notes:

The interaction between treatment status (i.e., dulaglutide and liraglutide) and patient characteristics (e.g., HbA1c ≥ 9% versus < 9%) was tested by including the interaction term in the mixed-model analysis.

*, **, and *** refer to *p*-values < 0.05, < 0.01, and < 0.001, respectively.

Appendix Figure S3. Flow chart of selection of studies included in the meta-analysis


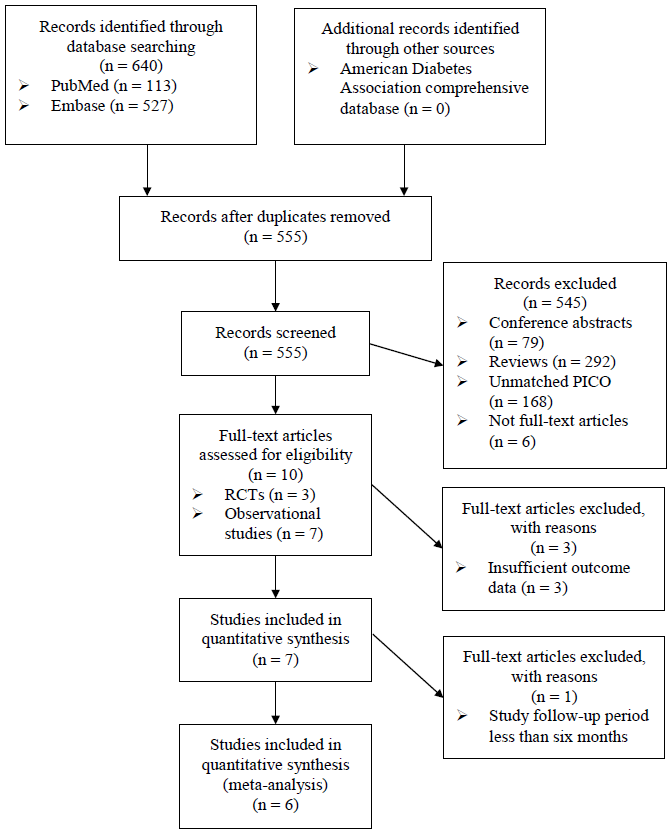


Abbreviation: PICO, patient intervention comparison outcome; RCTs, randomized control trials.

Appendix Table S4. Summary of existing studies that head-to-head compared dulaglutide and liraglutide

| Authors, year | Dungan et al. 2014 | Miyagawa et al. 2015 | Odawara et al. 2016 | Mody et al. 2018 | Brown et al. 2019 | Morieri et al. 2020 | This study |
| --- | --- | --- | --- | --- | --- | --- | --- |
| Country | Global | Japan | Japan | United States | Canada | Italy | Taiwan |
| Study design | RCT | RCT | RCT | Observational study | Observational study | Observational study | Observational study |
| Data source | - | - | - | Longitudinal administrative claims data | Multiple institutions in Canada | Multiple institutions in Italy | Multiple institutions in Taiwan |
| Period | 2012/06/20-2013/11/25 | 2012/04/01-2013/10/31 | 2012/04/01-2013/10/31 | 2014/11/01-2016/05/31 (follow to 2017/05/31) | 2017/04/01-2018/01/30 | 2010/01/01-2018/12/31 | 2016/01/01-2018/12/31 (follow to 2019/12/31) |
| Other therapies | Metformin | None | None | Other anti-hyperglycemic drugs | Other anti-hyperglycemic drugs | Other anti-hyperglycemic drugs | Other anti-hyperglycemic drugs |
| Sample size | 599 | 417 | 417 | 1,170 (1:1 matching) | 638 (1:1 matching) | 1,344 (1:1 matching) | 1,966 (1:1 matching) |
| Follow-up time | 6 months | 6 months | 12 months | 12 months | 6 months | 5.9 months | 12 months |
| Age | 56.6 | 57.4 | 57.4 | 53.5 | 57.6 | 61.7 | 57.1 |
| Sex (Men) | 48% | 81.7% | 81.7% | 50.1% | 57.3% | 64.5% | 47.9% |
| Baseline HbA1c | 8.1% | 8.12% | 8.12% | 8.77% | 8.4% | 8.2% | 9.35% |
| Baseline weight | 94.1 kg | 70.9 kg | 70.9 kg | NA | 97.9 kg | 96.6 kg | 77.7 kg |
| Baseline SBP | 131.5 mmHg | NA | NA | NA | 127.5 mmHg | 142.2 mmHg | 140.5 mmHg |

Abbreviations: RCT, randomized control trial; SBP, systolic blood pressure. NA: not available. “NA“ means that data/information was not available or indicated in the original paper.

Appendix Figure S4. Forest plot of 6-month difference in HbA1c between dulaglutide and liraglutide

(a)


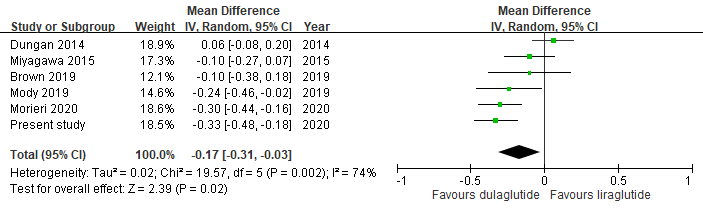


(b)


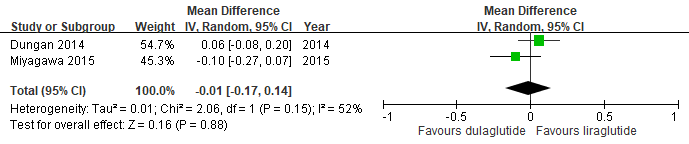


(c)


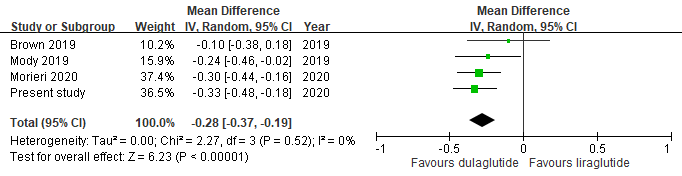


Legend: (a) All studies (including randomized controlled trials [RCTs] and observational studies), (b) RCTs only, and (c) observational studies only.

Appendix Figure S5. Forest plot of 12-month difference in weight loss between dulaglutide and liraglutide

(a)


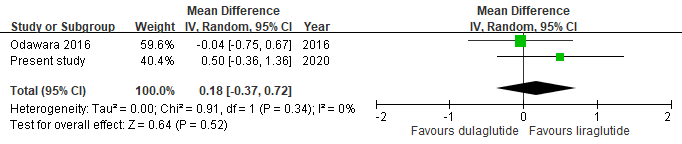


(b)


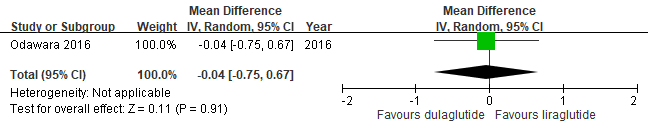


(c)


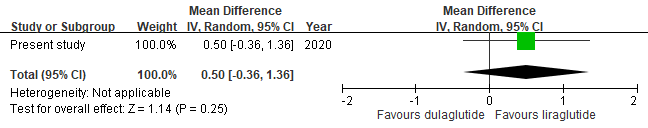


Legend: (a) All studies (including randomized controlled trials [RCTs] and observational studies), (b) RCTs only, and (c) observational studies only.

Appendix Figure S6. Forest plot of 6-month difference in weight loss between dulaglutide and liraglutide

(a)


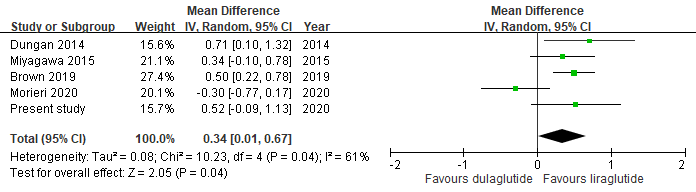


(b)


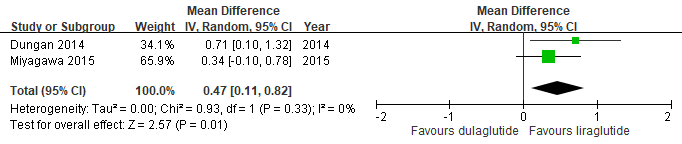


(c)


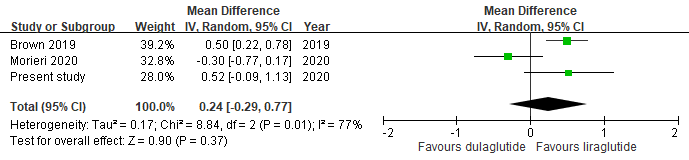


Legend: (a) All studies (including randomized controlled trials [RCTs] and observational studies), (b) RCTs only, and (c) observational studies only.

Appendix Figure S7. Forest plot of 12-month difference in systolic blood pressure change between dulaglutide and liraglutide

(a)


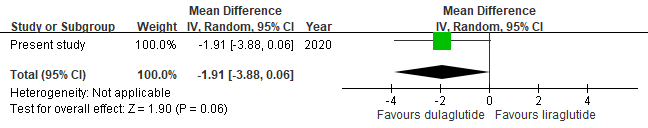


Legend: (a) All studies.

Appendix Figure S8. Forest plot of 6-month difference in systolic blood pressure change between dulaglutide and liraglutide

(a)


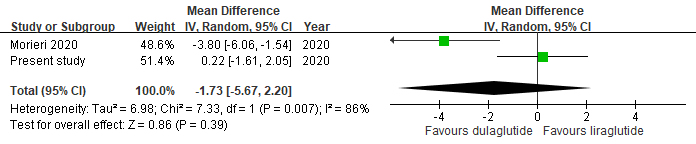


Legend: (a) All studies.
